# Supplementary material for: Single Amino Acid Mutations Affect Zika Virus Replication In Vitro and Virulence In Vivo
Source: Viruses. 2020 Nov 12;12(11):1295. doi: 10.3390/v12111295 (PMC7697975; doi:10.3390/v12111295)
Supplement: Supplementary file 1 [file viruses-12-01295-s001.pdf]

Suppl Table 1. Primers used for SDM

Mutant	Forward	Reverse
123	GCTATGGCAGTGGAGGTCACTAG	TGTGGTCAGCAGGAGGCC
894	ATGTGGAGAGCTCCACAGAGATTG	GGGGTTTTTACAGATCC
1404	CTGGGCCCATAGCCGCGGTCG	CCATCTCTATATCTGCCTTGGCGAACC
2074	CAACACCATACTGGAAGACAGTG	TTGGTCGTGCCATCAAAG
2086	GTGGACCAGATACGGAGAGAAAAGAG	ACCTCTGCCGGCACACTG

Suppl Table 2. Summary of Sanger sequencing data for detection of reversions of mutant to wild type amino acid.

Sequence data: Female mice			
Mutant	Reversions	Sample Types Tested	Reverted Type*
123	0/5	1 Placenta, 3 Spleen, 1 Blood	NA
894	0/5	3 Placenta, 2 Blood	NA
1404	0/5	1 Spleen, 4 Blood	NA
2074	1/5	3 Placenta, 2 Blood	Blood (1-T)
2086	4/6	3 Placenta, 1 Spleen, 2 Blood	Placenta (3-P) , Spleen (1-P)
**Sequence data: Male mice**			
Mutant	Reversions	Sample Types	Reverted Type*
123	0/12	3 Brain, 3 Spleen, 3 Testes, 3 Blood	NA
894	0/11	3 Brain, 3 Spleen, 3 Testes, 3 Blood	NA
1404	0/12	3 Brain, 3 Spleen, 3 Testes, 3 Blood	NA
2074	0/12	3 Brain, 3 Spleen, 3 Testes, 3 Blood	NA
2086	1/12	3 Brain, 3 Spleen, 3 Testes, 3 Blood	Spleen (1 -T)
**All Sequencing Data: 85 samples total (59 male, 26 female)**			
Mutant	Reversions	Sample Types	Reverted Type*
123	0/17	3 Brain, 3 Spleen, 3 Testes, 1 Placenta, 3 Blood	NA
894	0/16	3 Brain, 3 Spleen, 3 Testes, 3 Placenta, 5 Blood	NA
1404	0/17	3 Brain, 4 Spleen, 3 Testes, 7 Blood	NA
2074	1/17	3 Brain, 3 Spleen, 3 Testes, 3 Placenta, 5 Blood	Blood (1-T)
2086	5/18	3 Brain, 4 Spleen, 3 Testes, 3 Placenta, 5 Blood	Placenta (3-P), Spleen (1-P, 1-T)

* P = partial reversion; two peaks at mutation site; T = total reversion

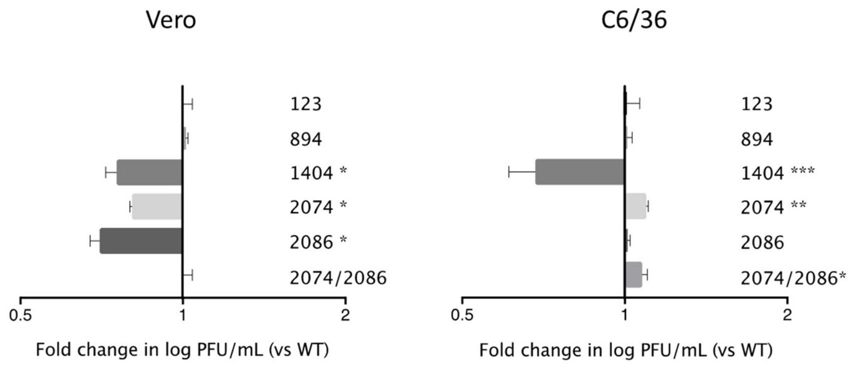

Suppl Fig. 1. Comparison of Host Viral Replication via Plaque Assay. (A) Synthetic mutant viral strains (123, 894, 1404, 2074, 2086, 2074/2086) are compared to the WT infectious clone in Vero (A, C) and C6/36 (B, D) cells provide evidence of differences in growth rates based on each point mutation. Significant differences are indicated by asterisks (*** = p <0.05; *** =p <0.001).**

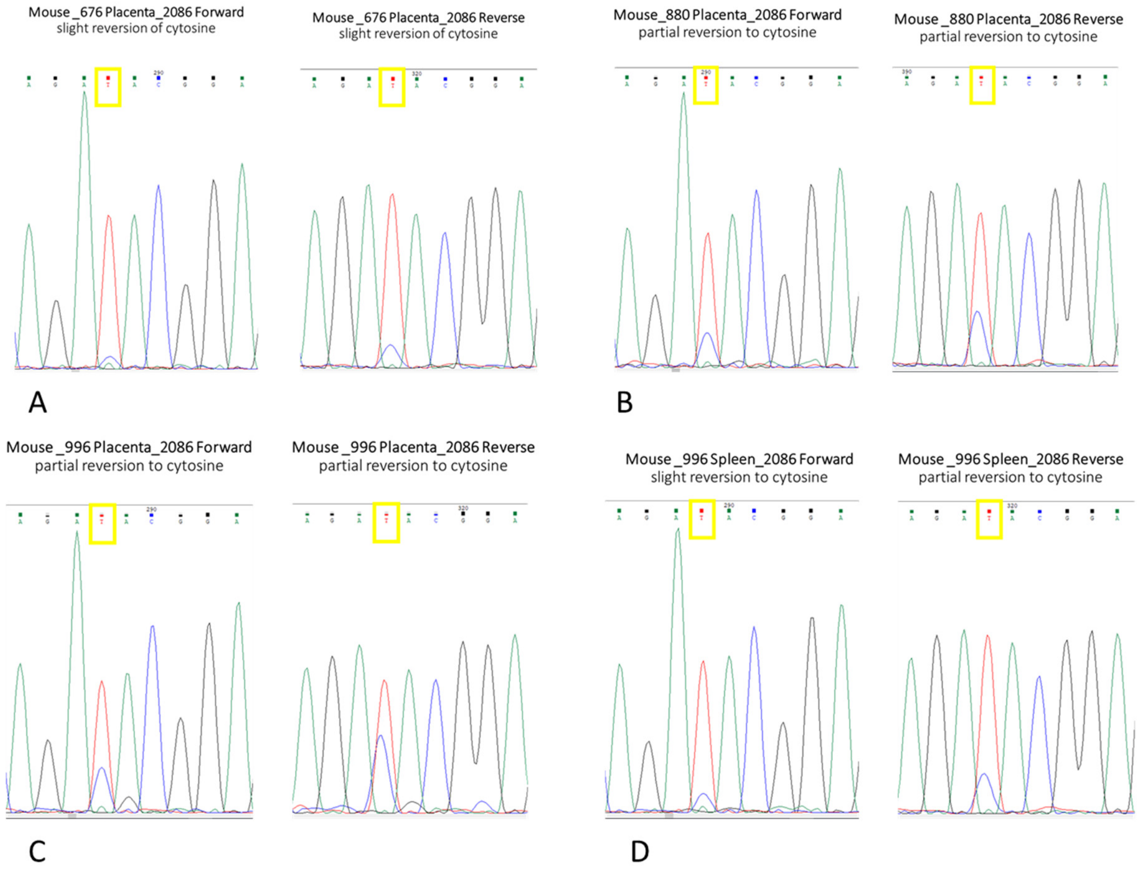

Suppl Fig. 2. Sanger sequence data showing partial reversions detected in mutant 2086 samples. For each set of traces, the mutated nucleotide is highlighted in a yellow box; mouse number, mutant number and tissue type is above sequencing traces. First set of peaks shown are sequencing data generated with the forward primer and second set is reverse complement data from the reverse primer. A) Mouse 676, mutant 2086, placenta; B) Mouse 880, mutant 2086, placenta; C) Mouse 996, mutant 2086, placenta; D) Mouse 996, mutant 2086, spleen.
